# Genomic scan of selective sweeps in Djallonké (West African Dwarf) sheep shed light on adaptation to harsh environments

**DOI:** 10.1038/s41598-020-59839-x

**Published:** 2020-02-18

**Authors:** Isabel Álvarez, Iván Fernández, Amadou Traoré, Lucía Pérez-Pardal, Nuria A. Menéndez-Arias, Félix Goyache

**Affiliations:** 1 Servicio Regional de Investigación y Desarrollo Agroalimentario, E-33394 Gijón, Spain; 2 Institut de l’Environnement et des Recherches Agricoles (INERA), Ouagadougou, 04 BP 8645 Burkina Faso; 3 CIBIO - InBIO, Universidade do Porto, 4485-661 Vairão, Portugal

**Keywords:** Population genetics, Functional genomics

## Abstract

The Djallonké (West African Dwarf) sheep is a small-sized haired sheep resulting from a costly evolutionary process of natural adaptation to the harsh environment of West Africa including trypanosome challenge. However, genomic studies carried out in this sheep are scant. In this research, genomic data of 184 Djallonké sheep (and 12 Burkina-Sahel sheep as an outgroup) generated using medium-density SNP Chips were analyzed. Three different statistics (iHS, XP-EHH and nSL) were applied to identify candidate selection sweep regions spanning genes putatively associated with adaptation of sheep to the West African environment. A total of 207 candidate selection sweep regions were defined. Gene-annotation enrichment and functional annotation analyses allowed to identify three statistically significant functional clusters involving 12 candidate genes. Genes included in Functional Clusters associated to selection signatures were mainly related to metabolic response to stress, including regulation of oxidative and metabolic stress and thermotolerance. The bovine chromosomal areas carrying QTLs for cattle trypanotolerance were compared with the regions on which the orthologous functional candidate cattle genes were located. The importance of cattle BTA4 for trypanotolerant response might have been conserved between species. The current research provides new insights on the genomic basis for adaptation and highlights the importance of obtaining information from non-cosmopolite livestock populations managed in harsh environments.

## Introduction

Drift and selection may cause selective sweep signatures on the genome, characterized by reduced diversity and extensive linkage disequilibrium. Favorable alleles increase in frequency more rapidly than expected for a neutral genetic scenario and further alter the allele frequencies of genomic variants near the beneficial mutation. The identification of signatures of selection has been carried out on several sheep breeds using genome-wide single nucleotide polymorphism (SNP) data^1^. Most analyses allowed to identify signatures of positive selection for economically important traits such as parasite resistance^2^, dairy production^3–5^, meat yield and growth^4,6,7^, fat metabolism and deposition^8–10^ or reproduction^11,12^. Other traits affecting breeds’ definition such as morphology, horn shape or coat color have caused selective sweeps on the sheep genome as well^6,12,13^.

However, there is an increasing interest in identifying selection signatures caused by human-mediated selection pressure for adaptation to different environmental conditions contrasting in climate (mainly precipitations and temperature), vegetation and altitude^10,14–17^. This approach allows to identify sheep populations (or breeds) more robustly suited to future climate changes and to obtain new insights on the history of the species after domestication.

Domestication and dissemination of sheep is a complex process, initiated about 11,000 years before present (yBP), which included different waves, specialization for different production goals and adaptation to different environmental conditions^18^. When compared with Northern Africa, the initial appearance of pastoralist communities based on small stock in Sub-Saharan West Africa may have been delayed by thousand years^19^. Reviewing the literature, Muiga and Hanotte^20^ suggested that domestic sheep entered into central Nile valley and central Sahara around 6,000 yBP reaching West Africa by 3,700 yBP. This lag in successful introduction of small ruminant livestock into West Africa stems from new animal diseases encountered by pastoral colonists entering biogeographic zones south of the Sahel, namely trypanosomiasis^19^. Native West African domestic ruminants (cattle, sheep and goat) share distinctive traits, namely trypanotolerance and small size. These species were subject to similar processes of adaptation to an extremely difficult environmental scenario and to trypanosome challenge^20^.

Djallonké (West African Dwarf) sheep^21,22^ are a small sized haired sheep population spread in the humid West and Central African regions. Djallonké sheep have the ability to maintain undepressed production and reproduction performance under parasite challenge even in the presence of a persistent parasitaemia. Therefore, they are considered resilient to trypanosome challenge^23^.

Most studies aiming at the identification of selective sweeps on the sheep genome have been carried out using highly structured populations including different cosmopolitan (commercial) and local sheep breeds^4,6,24^. Although there exist methodologies accounting for population structuring, such as hapFLK^25^, genetic heterogeneity amongst breeds carrying distinct mutations with similar phenotypic effects may lead selective sweeps to not reach statistical significance, particularly when sample size is moderate^4^.

In this research, genomic data generated using medium-density Chips on a sample of Burkina Faso Djallonké sheep were assessed to identify candidate selection sweep regions spanning genes associated with different biological pathways putatively involved in the adaptation of sheep to the environmental conditions of the humid areas of West Africa. The genomic profile of this population, compared with that of a small sample of Burkina-Sahel sheep when necessary, was explored for signatures of selection using various Extended Haplotype Homozygosity (EHH-)based statistics^26^. The corresponding cattle orthologous genomic regions were compared with the bovine chromosomal areas in which Quantitative Traits Loci (QTL) for trypanotolerance–related traits have been reported^27^.

## Methods

### Sample used

A total of 184 DNA samples of Djallonké lambs (64 males and 120 females) previously analyzed using microsatellites^28^ were available. Up to 166 individuals were sampled in the surroundings of Mangodara (latitude 9°53′59.99″N; longitude 4°20′59.99″W; Comoé province), southern Burkina Faso. The other 18 individuals (9 males and 9 females) were sampled in Dédougou (latitude 12°27′48.17″N; longitude 3°27′38.7″W; Mouhoun province), southwestern Burkina Faso. Both sampling areas belong to the environmental Sudan-Guinea Savannah humid region (tse-tse challenged) of Burkina Faso^21,22^. The Sudan-Guinea Savannah environmental region has annual rainfall higher than 900 millimeters (with precipitations in Mangodara and Dédougou varying from 1,000 to 1,200 millimeters per year), predominance of woodlands and savannahs, and temperatures varying from 19 °C to 36 °C^29^. Additionally, 12 individuals belonging to the Burkina-Sahel sheep breed were sampled in the surroundings of Dori (latitude 14°02′7.44″N; longitude 0°02′4.20″E; Séno Province), located on the arid environmental Sahel region of Northern Burkina Faso, to be used as an outgroup. The Sahel environmental region has less than 600 millimeters of rainfall per year, temperatures varying from 15 °C to 43 °C and bushy vegetation^29^. Morphological and genetic description of both the Djallonké and the Burkina-Sahel sheep populations of Burkina Faso is available in the literature^21,22,30^. The management of the Djallonké sheep of Burkina Faso has been described as well^31^. Sheep breeding in Burkina Faso is based on small-holders and carried out in extensive conditions with no supplementation. In the Sudan-Sahel environmental area, animals perform communal grazing of native pasture with no restrictions during the dry season and about 12 hours a day during the rainy season. In the Sahel area, management system is traditional and extensive with little or no management inputs: shelter or minerals. Sheep are managed by their owners (Fulani ethnic group). Communal grazing is less frequent. In all cases, use of fodder crops and conserved forages is negligible. Planned matings are not usual.

### SNP genotyping and quality control

The whole dataset was typed using the Ovine 50 K SNP BeadChip following standard protocols (http://www.illumina.com). The software GenomeStudio (Illumina Inc., San Diego, CA) was used to generate standard.ped and map files. Sample and marker-based quality control measures were performed using the program PLINK V 1.09^32^. GenCall score cutoff of 0.15 and average sample call rate of 99% were considered. All unmapped SNPs, those mapping to sexual chromosomes, SNPs with a genotyping rate lower than 90% or those below a Minor Allele Frequency threshold of 0.05 were removed. To avoid departures from Hardy-Weinberg proportions due to genotyping errors, SNPs that did not pass Hardy-Weinberg test for P ≤ 0.001 were removed as well. A total of 46,977 SNPs located on the 26 ovine autosomes passed the quality control for the whole sample analyzed.

### Diversity and population structuring analyses

Genetic diversity of the available population was assessed using the software VCFtools^33^. Within subpopulation (Djallonké and Burkina-Sahel) inbreeding and between subpopulations F_ST_ were computed.

A clustering analysis was carried out using the program Admixture v1.23^34,35^ which calculates maximum likelihood estimates of individual ancestries based on data provided by multiple loci. Analyses were conducted for 1 ≤ K ≤ 8 being K the number of clusters given the data. The optimal number of clusters was determined via cross-validation as the value of K exhibiting the lower cross-validation error compared to other K values. Data set was divided into 5 folders for each K. Folders were sequentially used as test sets while the other four were used for training.

The program PLINK V 1.09^32^ was used to compute Principal Component Analysis (PCA). Eigenvectors computed for each individual were used to construct dispersion plots accounting for the Admixture Cluster to which individuals were assigned. The 75% confidence regions of the relationships among individuals within cluster were illustrated using contour plots. Plots were constructed using the library ggplot2 of R (http://CRAN.R-project.org/).

### Identification of selective sweeps

Three complementary EHH-based statistics, integrated Haplotype Score^36^ (iHS), XP-EHH^37^ and nSL^38^, were used to assess genome-wide signatures of selection in Djallonké sheep. These three approaches were proposed to overcome the main concern affecting the original EHH test^26^ i.e. the yield of a high number of false positives due to the strong influence on results of the demographic history of the population under studied.

The iHS test compares the EHH between derived and ancestral alleles within a population searching for loci where the derived allele resides on a longer haplotype than the ancestral allele^36^. In this way, the iHS values are less affected by the demographic history of the population and are more suitable for identifying incomplete sweeps, where the selected allele is not fixed in the sample.

The XP-EHH test compares the frequencies of the selected haplotypes between two populations to detect ongoing or nearly fixed selection signatures^37^. Therefore, the XP-EHH test is more robust than the EHH statistics in scenarios of complete selective sweeps. This test has an increased power to detect selection signatures using small sample sizes and allows to use groups of genetically similar populations.

In contrast to iHS and XP-EHH, which measures the length of haplotypes in terms of genetic distance and thereby uses recombination, the nSL statistics^38^ measures haplotype lengths in terms of the number of segregating sites in the sample, making it more robust to recombination rate variations. In addition, although both iHS and nSL target at the identification of incomplete sweeps, the nSL statistic has improved power in detecting soft sweeps^38^.

Both large positive and negative iHS, XP-EHH and nSL scores were considered potentially informative. Although negative iHS scores would mean that a derived allele would have swept up in frequency and, therefore, could be considered of more interest, large positive values can indicate that ancestral alleles hitchhike with the selected site^39^. Furthermore, scenarios in which selection switch to favor an ancestral allele cannot be discarded. Similar rationale can be applied to the recombination-free counterpart of iHS, the nSL test. In the case of XP-EHH scores large positive and negative values would identify selection events in the studied population and in the reference population, respectively. Both cases are informative in scenarios of poor differentiation.

All estimates were carried out using the program *selscan* v.1.0.4^40^, freely available at http://github.com/szpiech/selscan, fitting the parameters recommended by the authors: maximum EHH extension in bp (“–max-extend” option) 1000000, maximum gap allowed between two SNPs in bp (“–max-gap” option) 200000, EHH decay cutoff (“–cutoff” option) 0.05. Whatever the statistics computed, the output results for each SNP were frequency-normalized over all chromosomes using the program *norm*, provided with *selscan*. This normalization was carried out using default parameters as well: number of frequency bins (“–bins” option) 100. As implemented by default in the program *norm*, normalized values higher than |2| were used to identify SNPs under selection.

The program *selscan* is ‘dumb’ with respect ancestral/derived alleles, simply assigning the “reference” allele depending on previous arbitrary allele coding^40^. Unstandardized iHS scores are then computed as ln(iHH1/iHH0)^40^, being iHH the integrated haplotype homozygosity for the ancestral (0) and derived (1) haplotypes. There is empirical evidence suggesting that iHS values obtained from a given subset of SNPs with or without ancestral allele information (i.e. random assignment) are highly consistent^41^. Therefore, this has been proved to be a reliable strategy.

Previous studies aiming at the ascertainment of the genomic diversity in Djallonké sheep used as reference population Sahel sheep sampled in the same geographical area^42^. Therefore, Burkina-Sahel individuals were used for the XP-EHH analyses.

Candidate selection sweep regions were defined starting from: a) individual SNPs identified as being under selection by at least two of the statistics applied; and b) adjacent SNPs identified as being under selection by at least two different statistics. Two SNPs were considered adjacent if their 75 kb up- or down-stream regions overlapped. All other SNPs identified as being under selection by any statistics with their 75 kb up- or down-stream regions sequentially intersecting with the 75 kb regions surrounding the SNPs selected using the strategies a) and b) described above were assigned to the corresponding selection sweep region.

### Functional characterization of the candidate regions

Candidate genes were considered if their boundaries fell within 75 kb up- or down-stream the selection sweep regions defined. Protein-coding genes found within the candidate regions were retrieved from the Ensembl Genes 91 database, based on the Oar v3.1 ovine reference genome (http://www.livestockgenomics.csiro.au/sheep/oar3.1.php) using the BioMart tool^43^. The Ensembl Genes 91 database and the BioMart tool were also used to identify the corresponding orthologous cattle genes, based on the UMD3.1 bovine reference genome. All the identified genes were processed using the functional annotation tool implemented in DAVID Bioinformatics resources 6.8^44^ to determine enriched functional terms. An enrichment score of 1.3, which is equivalent to the Fisher exact test P-value of 0.05, was used as a threshold to define the significantly enriched functional terms in comparison to the whole bovine reference genome background. Relationships among genomic features in different chromosome positions were represented using the R software package shinyCircos^45^.

### Overlap with cattle trypanotorelance-related QTLs

The bovine QTLs for trypanotolerance–related traits^27^ which were mapped on the bovine Btau 4.0 reference genome assembly were downloaded from the cattle QTL database (http://www.animalgenome.org/cgi-bin/QTLdb/BT/index). The QTL genome coordinates were then re-mapped on the bovine UMD 3.1 reference genome assembly using the NCBI genome remapping online service (https://www.ncbi.nlm.nih.gov/genome/tools/remap). The intersectBed function of the BedTools software^46^ was used to overlap these trypanotolerance-related QTLs with the bovine regions on which the orthologous functional ovine candidate genes identified were located.

## Results

### Genetic diversity and structuring of data

Mean inbreeding (and standard deviation) in Djallonké sheep was of 0.058 ± 0.070. This was higher than the value computed for the Burkina-Sahel sample (0.014 ± 0.020). Differentiation between Djallonké and Burkina-Sahel sheep, assessed computing weighted Weir and Cockerham’s F_ST_, was 0.066.

**Figure 1:**
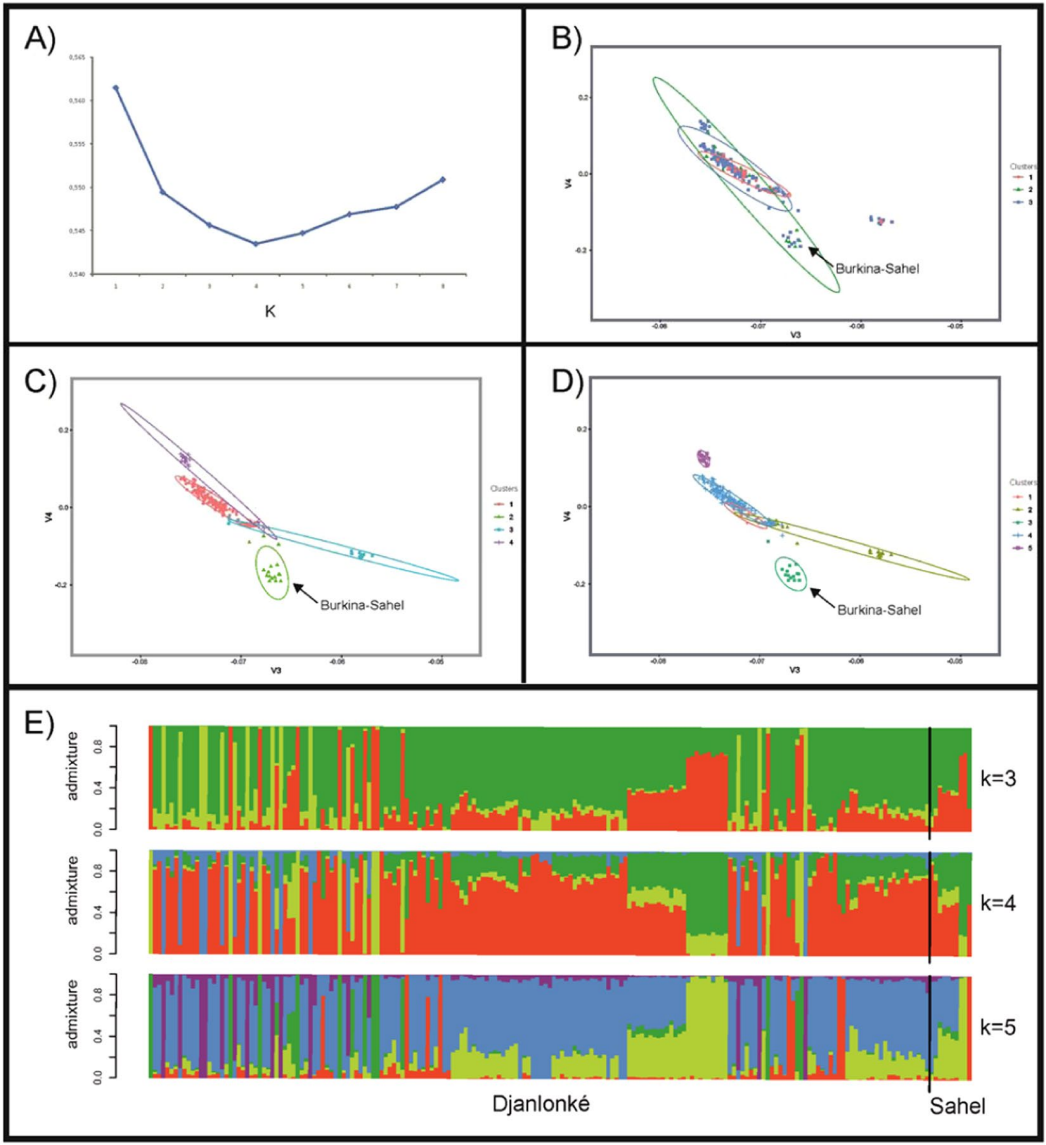
Plots showing cross-validation errors for each K tested using the program Admixture v1.23, with the lowest error for K = 4 (Plot **A**), and dispersion of the Djallonké and Burkina–Sahel individuals according the two first factors computed via PCA (Plots **B**, **C** and **D**). Factors 1 and 2 explained 3.2% and 2.4% of the total variance, respectively. Contour plots illustrate the 75% confidence region of the relationships among the individuals assigned to each Cluster identified using Admixture v1.23 for K = 3 (Plot B), K = 4 (Plot C) and K = 5 (Plot **D**). The location of most Burkina-Sahel individuals is highlighted Furthermore, barplots of individual ancestries estimated using Admixture v1.23 for K = 3, K = 4 and K = 5 are shown (Plot **E**).

The results of the admixture analysis informed that the lowest cross-validation error was at K = 4 (Fig. 1). However, differences between cross-validation errors for K = 3, K = 4 and K = 5 were lower than 0.005. Most Djallonké individuals (144 for K = 3, 145 for K = 4 and 140 for K = 5) grouped into a single Cluster. However, assignment of the Burkina-Sahel individuals into a given Cluster was not clear. No differentiation was found between Djallonké sheep sampled either in Mangodara or Dédougou. Most Dédougou samples (from 75% for K = 5 to 100% for K = 3) clustered within the main group formed by Mangodara samples.

Furthermore, most Burkina-Sahel individuals (10) clustered with Djallonké individuals for K = 3 (Fig. 1B). Although Burkina-Sahel individuals tended to form particular Clusters for K = 4 and K = 5 (Figs. 1C,D), they shared these Clusters with a number of Djallonké individuals for K = 4 (4) and for K = 5 (7).

PCA identified 19 factors with eigenvalue higher than 1 which accounted for a total of 25.1% of the genetic variance. Figure 1 shows the dispersion of the individuals and the 75% confidence region of the relationships between individuals assigned to each Cluster identified using Admixture v1.23. Independently of the number of K considered, PCA did not allow to assess a clear separation of the individuals. This was particularly remarkable for K = 3 (Fig. 1B) in which the 75% confidence region of dispersion of the individuals assigned to each of the three clusters overlapped. Although this did not happen for K = 4 and K = 5, PCA illustrated a weak structure in the population analyzed: a significant number of individuals assigned to different clusters were highly intermingled in the middle of the plots.

### Candidate regions under selection

**Figure 2:**
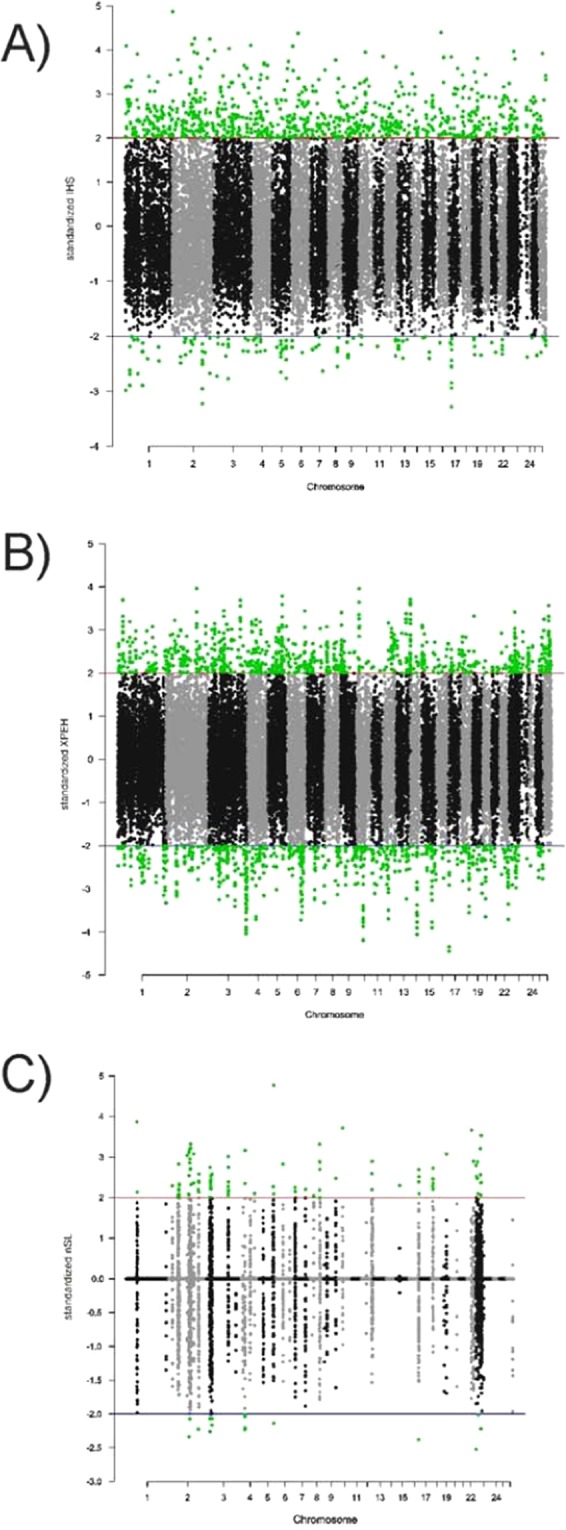
Manhattan plots summarizing results corresponding to iHS (Plot **A**), XP-EHH (Plot **B**) and nSL (Plot **C**) analyses. SNPs with normalized values higher than |2| are highlighted in green.

Figure 2 shows the Manhattan plots illustrating the SNPs identified as being under selection pressure on all ovine autosomes according to the three tests assayed. After normalization 974 SNPs (Supplementary Table S1), 1,633 (Supplementary Table S2) and 110 (Supplementary Table S3) SNPs were considered to be under selection using the iHS, XP-EHH and nSL tests, respectively.

After crossing the output of the three tests applied, a total of 207 candidate selection sweep regions on all autosomes, except for OAR20 and OAR21, were defined (Table 1; Supplementary Table S4). These regions comprised a total of 34,957,703 bp and 555 different SNPs. Up to 153 SNPs were identified as being under selection by two different statistics. Four chromosomes (OAR1, 2, 3 and 23) gathered 46% of the candidate selection sweep regions.

**Table 1:** Summary of the candidate selection sweep regions identified per ovine chromosome (OAR).

OAR	N	Length	n
1	18	3549100	67 (10)^a^
2	34	6581006	44 (34)
3	27	5892878	105 (19)
4	16	2450404	45 (12)
5	8	1845327	27 (10)
6	10	2590704	43 (5)
7	4	521393	11 (1)
8	9	1097043	17 (5)
9	5	983744	17 (2)
10	4	1077755	16 (9)
11	2	116626	2 (2)
12	7	822135	16 (4)
13	7	960058	18 (3)
14	1	57444	2 (0)
15	6	914153	21 (3)
16	6	181219	7 (3)
17	4	578619	9 (4)
18	10	1669082	27 (7)
19	2	153579	3 (1)
20			
22	3	486195	7 (2)
23	17	1407368	34 (11)
24	2	286693	3 (2)
25	1	107671	2 (2)
26	4	627507	12 (2)
Totals	207	34957703	555 (153)

The number of selection sweeps identified (N), the sum of their lengths (in bp) and the sum of the SNPs defining them (n) are given per chromosome.

^a^sum of the SNPs identified by two different EHH-based statistics.

### Identification of functional candidate genes linked to selection sweeps

Gene-annotation enrichment analysis allowed to identify a total of 491 potential candidate ovine genes in the 75 kb up- and down-stream regions surrounding the selection sweep regions defined previously. A full description of these 491 ovine genes, including their identification, description and location is given in Supplementary Table S5. Details on the corresponding orthologous cattle genes are given in Supplementary Table S5 as well.

**Figure 3:**
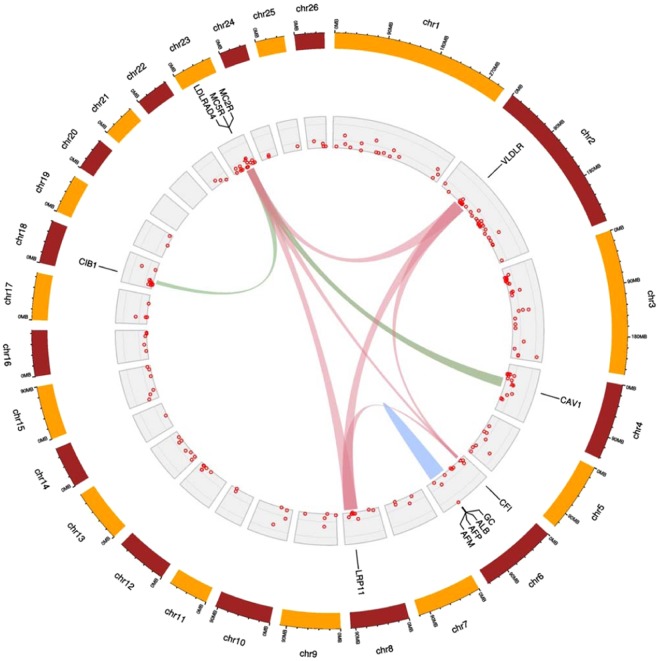
Circular map, created using the ShinyCircos^45^ software, summarizing information on Functional Clusters identified using selection sweeps in Djallonké sheep. Chromosomes are shown in the outermost circle. The innermost circles show the distribution of selection sweeps within a chromosome. Differences in height among selection sweeps inform on the number of SNPs defining them. Candidate genes forming the significantly enriched functional term clusters are indicated besides their genomic localization. At the center of the map, links among candidate genes belonging to the same functional cluster are illustrated using the same color.

Functional annotation conducted on these genes allowed to identify 22 different functional term clusters (Supplementary Table S6). However, only three functional term clusters were significantly enriched (enrichment score higher than 1.3; Table 2; Fig. 3). Functional Cluster 1 (enrichment score = 3.24) included four genes (*ALB*, *GC*, *AFP* and *AFM*) involved in transport functions of the plasma membrane and homeostasis. Functional Cluster 2 (enrichment score = 1.39) included four genes (*MC2R*, *CIB1*, *MC5R* and *CAV1*) involved in angiogenesis, answer to ischemia and hypoxia. Functional Cluster 3 (enrichment score = 1.31) included four genes (*LDLRAD4*, *LRP11*, *CFI* and *VLDLR*) involved in lipid metabolism and response to temperature stress.

**Table 2:** Significantly enriched functional term clusters (enrichment score higher than 1.3) for genes identified within the 75 kb regions flanking the candidate selection sweeps identified using genome-wide iHS, XP-EHH and nSL statistics following DAVID analysis.

Functional Cluster (enrichment score)	Category	Term and Description	Genes	P-Value	Fold Enrichment
Cluster 1 (3.24)	INTERPRO	IPR014760:Serum albumin, N-terminal	ENSOARG00000013782 (*ALB*), ENSOARG00000012835 (*GC*), ENSOARG00000013966 (*AFP*), ENSOARG00000014129 (*AFM*)	8.61E-05	38.40
	INTERPRO	IPR000264:ALB/AFP/VDB	ENSOARG00000013782 (*ALB*), ENSOARG00000012835 (*GC*), ENSOARG00000013966 (*AFP*), ENSOARG00000014129 (*AFM*)	8.61E-05	38.40
	INTERPRO	IPR020857:Serum albumin, conserved site	ENSOARG00000013782 (*ALB*), ENSOARG00000012835 (*GC*), ENSOARG00000013966 (*AFP*), ENSOARG00000014129 (*AFM*)	8.61E-05	38.40
	SMART	SM00103:ALBUMIN	ENSOARG00000013782 (*ALB*), ENSOARG00000012835 (*GC*), ENSOARG00000013966 (*AFP*), ENSOARG00000014129 (*AFM*)	8.81E-05	37.89
	INTERPRO	IPR020858:Serum albumin-like	ENSOARG00000013782 (*ALB*), ENSOARG00000012835 (*GC*), ENSOARG00000013966 (*AFP*), ENSOARG00000014129 (*AFM*)	1.70E-04	32.00
	PIR_SUPERFAMILY	PIRSF002520:serum albumin	ENSOARG00000013782 (*ALB*), ENSOARG00000013966 (*AFP*), ENSOARG00000014129 (*AFM*)	1.19E-03	47.29
	INTERPRO	IPR021177:Serum albumin/Alpha-fetoprotein	ENSOARG00000013782 (*ALB*), ENSOARG00000013966 (*AFP*), ENSOARG00000014129 (*AFM*)	2.51E-03	36.00
	GOTERM_BP_DIRECT	GO:0006810~transport	ENSOARG00000013782 (*ALB*), ENSOARG00000013966 (*AFP*), ENSOARG00000014129 (*AFM*)	4.33E-01	2.04
Cluster 2 (1.39)	INTERPRO	IPR023415:Low-density lipoprotein (LDL) receptor class A, conserved site	ENSOARG00000003950 (*MC2R*), ENSOARG00000002239 (*MC5R*), ENSOARG00000001337 (*CAV1*)	1.70E-02	12.47
	SMART	SM00192:LDLa	ENSOARG00000003950 (*MC2R*), ENSOARG00000002239 (*MC5R*), ENSOARG00000001337 (*CAV1*)	5.20E-02	8.08
	INTERPRO	IPR002172:Low-density lipoprotein (LDL) receptor class A repeat	ENSOARG00000003950 (*MC2R*), ENSOARG00000012128 (*CIB1*), ENSOARG00000002239 (*MC5R*), ENSOARG00000001337 (*CAV1*)	7.59E-02	4.04
Cluster 3 (1.31)	INTERPRO	IPR023415:Low-density lipoprotein (LDL) receptor class A, conserved site	ENSOARG00000002144 (*LDLRAD4*), ENSOARG00000002898 (*LRP11*), ENSOARG00000005291 (*CFI*), ENSOARG00000013303 (*VLDLR*)	3.83E-02	5.33
	SMART	SM00192:LDLa	ENSOARG00000002144 (*LDLRAD4*), ENSOARG00000002898 (*LRP11*), ENSOARG00000005291 (*CFI*), ENSOARG00000013303 (*VLDLR*)	5.10E-02	4.74
	INTERPRO	IPR002172:Low-density lipoprotein (LDL) receptor class A repeat	ENSOARG00000002144 (*LDLRAD4*), ENSOARG00000002898 (*LRP11*), ENSOARG00000005291 (*CFI*), ENSOARG00000013303 (*VLDLR*)	5.96E-02	4.46

A description of the genes involved in the definition of these three functional clusters is given in Table 3. The four genes forming Functional Cluster 1 were located on OAR6 and in close vicinity spanning a chromosomal area of 87,639 bp (from position 88,136,611 to position 88,224,250). Functional Cluster 3 included gene *CFI* which was located on OAR6 as well, although on a distant area. Functional Clusters 2 and 3 had neighboring genes located on OAR23 (genes *MC2R* and *MC5R* for Cluster 2 and gene *LDLRAD4* for Cluster 3) on an area spanning 247,960 bp.

**Table 3:** Description of the sheep genes included in the three functional clusters displaying enrichment score higher than 1.3.

Functional	Gene		Sheep gene information				Orthologous cattle gene information			
Cluster	name	description	EnsemblID	OAR	start	end	Cattle EnsemblID	BTA	start	end
Cluster 1	*ALB*	albumin [Source:NCBI gene;Acc:443393]	ENSOARG00000013782	6	88136611	88159187	ENSBTAG00000017121	6	90232762	90251126
	*GC*	GC, vitamin D binding protein [Source:HGNC Symbol;Acc:HGNC:4187]	ENSOARG00000012835	6	86619919	86657661	ENSBTAG00000013718	6	88695940	88739180
	*AFP*	alpha fetoprotein [Source:HGNC Symbol;Acc:HGNC:317]	ENSOARG00000013966	6	88166794	88190190	ENSBTAG00000017131	6	90258976	90280522
	*AFM*	afamin [Source:HGNC Symbol;Acc:HGNC:316]	ENSOARG00000014129	6	88198267	88224250	ENSBTAG00000047833	6	90291078	90312046
Cluster 2	*MC2R*	melanocortin 2 receptor [Source:NCBI gene;Acc:443482]	ENSOARG00000003950	23	43893855	43894748	ENSBTAG00000011038	24	44006264	44024553
	*CIB1*	calcium and integrin binding 1 [Source:NCBI gene;Acc:100141300]	ENSOARG00000012128	18	20723490	20727112	ENSBTAG00000021275	21	22043354	22046525
	*MC5R*	Ovis aries melanocortin 5 receptor (MC5R), mRNA. [Source:RefSeq mRNA;Acc:NM_001078656]	ENSOARG00000002239	23	43867867	43870160	ENSBTAG00000009143	24	43983880	43984857
	*CAV1*	caveolin 1 [Source:NCBI gene;Acc:445468]	ENSOARG00000001337	4	51786039	51820791	ENSBTAG00000017869	4	52173110	52208687
Cluster 3	*LDLRAD4*	low density lipoprotein receptor class A domain containing 4 [Source:HGNC Symbol;Acc:HGNC:1224]	ENSOARG00000002144	23	43646788	43801055	ENSBTAG00000009139	24	43861727	43919241
	*LRP11*	LDL receptor related protein 11 [Source:VGNC Symbol;Acc:VGNC:30988]	ENSOARG00000002898	8	73814860	73836141	ENSBTAG00000032007	9	88115145	88183314
	*CFI*	complement factor I [Source:NCBI gene;Acc:101119307]	ENSOARG00000005291	6	15708801	15753015	ENSBTAG00000034501	6	16765034	16815352
	*VLDLR*	very low density lipoprotein receptor [Source:HGNC Symbol;Acc:HGNC:12698]	ENSOARG00000013303	2	70468444	70486178	ENSBTAG00000018517	8	42109679	42141155

Besides the gene name and description, the following information is provided: the identification of the gene retrieved from the Ensembl Genes 91 database, the ovine chromosome (OAR) on which the gene is located and the positions (in bp) of start and end of the gene within the chromosome. The same information is given for the orthologous cattle genes.

### Correspondence with cattle trypanotolerance-related QTLs

The relationship between the orthologous cattle genes listed in Table 3 and the cattle QTLs reported for trypanotolerance-related traits^27^ are given in Supplementary Table S7. Only the cattle gene *CAV1*, assigned to Functional Cluster 2, intersected with a QTL (QTL ID no. 10515) reported on BTA4 for trypanosomes’ load.

## Discussion

As expected^22,47^, the population analyzed is poorly structured. This is true even if the Burkina-Sahel individuals are considered (Fig. 1). This situation is consistent with the more common West African livestock scenario in which no artificial selection programmes exist and long-distance livestock trading is intense.

Although Sahelian and Djallonké sheep show large morphological differences and are expected to have different ancestral origins^21,22^, the Burkina-Sahel sheep do not show a clear separation from the Djallonké individuals (Fig. 1). At the neutral loci level, it is assumed that genetic differentiation in West African livestock ruminant populations is basically due to geographic distance^47,48^. Morphology, production goals or differences in origin do not result in high genetic differentiation. The poor genetic structuring identified may be due to different unobserved founder events underlying data as a consequence of a very traditional management system in which unplanned natural matings are the rule^28^.

In scenarios of poor differentiation amongst individuals, the use of EHH-based tests to identify genomic areas under selection is preferable to F_ST_-based methods which need at least two different populations in dataset^1^. We further tested this running the software BayeScan v. 2.1^49^ on our Djallonké genotypes. Only four SNPs (OAR1_22330907.1, OAR12_17378202.1, s56240.1 on OAR14 and OAR16_10423797.1) were identified as being under diversifying selection (positive α values). The inclusion of the Burkina-Sahel individuals in the analyses allowed to identify ten SNPs under diversifying selection (Supplementary Table S8). However, the set of SNPs listed above and their 75 kb up- or down-stream regions did not overlap with the genomic regions surrounding the SNPs identified using EHH-based tests.

The 207 selection sweep regions identified in Djallonké sheep spanned about 35 Mbp which is about 1.3% of the roughly 2.65 Gbp covered by SNPs typed. These figures are consistent with those reported in previous works using similar conservative criteria in the definition of selection signatures. The concordant genomic regions identified using two different EHH-based statistics on three different sheep breeds (Sunite, German Mutton and Dorper) typed with the same SNP Chip used here spanned chromosomal regions summing up from 1,049,753 bp (Dorper) to 1,507,244 bp (Sunite)^24^. The analysis of the genomic profile, using three different methods (F_ST_, iSH and RsB), of three Brazilian sheep breeds gave only 5 (out of 246) coincident selection signatures^12^. When only two tests were considered the number of coincident selection signatures was 37^12^.

### Biological importance of the functional clusters identified

Gene-annotation enrichment analysis carried out allowed to identify various functional term clusters involved in signaling pathways associated directly or indirectly with environmental adaptation, such as control of metabolic stress, homeostasis, modulation of immune and inflammatory responses, cell proliferation and migration (Supplementary Table S6). The statistically significant Functional Clusters identified depicted particular genetic aspects of adaptation to harsh environments. Although some of the genes included in such Functional Clusters are involved in immune response (e.g. *GC* and *CFI* genes), they are mainly related to metabolic response to stress.

Functional Cluster 1 included four genes (*ALB*, *GC*, *AFP* and *AFM*) belonging to the family of albumin genes. Because of their location at the same chromosome locus and in the same transcriptional orientation across species, they are proposed to originate from common predecessor and to be cooperatively regulated^50^. Albumin (*ALB*) is involved on transport of metals, fatty acids, cholesterol, bile pigments, and drugs. It is a key element in the regulation of osmotic pressure and distribution of fluid between different compartments. In general, albumin represents the major and predominant antioxidant in plasma, a body compartment known to be exposed to continuous oxidative stress^51^. Variants of the albumin genes family, including the fetal counterpart of serum albumin (*AFP*) and afamin/alpha-albumin (*AFM*) genes, are associated with the development of the metabolic syndrome in adult humans^52,53^. In turn, the *GC* gene is part of the complement system, a key component of innate immunity and susceptibility to diseases with a major role in tissue homeostasis, degeneration, and regeneration^54^. The *GC* gene has major functions including the modulation of immune and inflammatory responses via regulation of chemotaxis and macrophage activation, transport by binding of fatty acids in collaboration with albumin and control of bone development^55^.

Functional Cluster 2 included two genes (*MC2R* and *MC5R*) encoding proteins belonging to the G protein-coupled receptors family involved in the response to stress via binding plasma adrenocorticotropin hormone (ACTH). While the *MC2R* gene acts on adrenal steroidogenesis and regulation of the glucocorticoid axis, the *MC5R* gene regulates the function of sebaceous glands with effect on water repulsion and thermoregulation^56^. In turn, the *CIB1* gene encodes a protein involved in cell proliferation, particularly proplatelets, therefore acting on thrombopoiesis^57^, while the *CAV1* gene has a critical role in signal transduction and trafficking for its interplay with steroid receptors and is associated with the metabolic syndrome in humans^58^.

Genes assigned to Functional Cluster 3 have a major role on response to stress and immunity. The *LDLRAD4* gene encodes a protein involved in the regulation of the transforming growth factor- ß (TGF-ß) signaling^59^ which plays a pivotal role in cell differentiation, apoptosis, cell migration, production of matrix proteins, angiogenesis, and anti-proliferative responsiveness. The *VLDLR* gene is also involved in the regulation of cell proliferation and migration^60^. Another low density lipoprotein receptor gene belonging to this Cluster (*LPR11*) is associated with chronic stress caused by food or water deprivation or elevated or cold temperature^61^. Finally, the complement factor I (*CFI*) gene encodes a serine protease forming part of the complement system, which is involved in innate and adaptive immune responses for prevention and control of diseases^62^.

### General discussion on adaptation to West African environment

The genomic regions harboring candidate genes of functional importance identified in the current study are, in general, different to those previously reported in the literature. This is not surprising in the case of works aiming at the characterization of the effect on the sheep genome of directional selection for dairy^3,5^ or meat traits^6,7^. However, interestingly enough, it also occurred when reports aiming at the identification of genomic signals of adaptation to extreme environments were considered. It is worth pointing out that most studies in this field aimed at the identification of genomic areas related to adaptation to high altitude^10,17^ or to high temperature in basically arid environments^15,63^. Such works involved several sheep populations bred in different geographical areas highly contrasting in environments, production systems and goals^14,15,63^. Therefore, the ability to identify selection signatures related to thermotolerance or hypoxia may be affected by the influence of others more likely linked to production or reproduction performance.

The Djallonké population analyzed here is a homogeneous population managed in a harsh, hot and humid environment subject to different disease challenges, namely trypanosomosis^23^. Although it is assumed that genes related to immune response and thermotolerance are basic for environmental adaptation, the lack of concordance between the genomic signals identified here and those previously reported would suggest that the genomic areas involved in resistance to hot climate stress may be different if they are identified in populations bred either in humid or arid areas. In this respect, the genomic areas conserved between sheep and goat indigenous to the hot arid environment of Egypt^63^ are completely different than those identified as functionally important in Djallonké sheep. In any case, it cannot be discarded that the history of the population is affecting results. The only coincidence between our study and others in the literature is a selection signature on OAR23 carrying out genes *MC5R* and *MC2R* in Ethiopian Menz and Red Maasai breeds (East African sheep)^13^. While the Ethiopian Menz is a fat-tailed coarse wooled sheep probably descending from very ancient importations from Arabia (http://eth.dagris.info/node/2448), the Red Maasai is a hair sheep like Djallonké, although medium to large body sized, with well known resistance to gastrointestinal parasites^64^.

Trypanosome challenge has been hypothesized to be a major historical force influencing the formation of native West African domestic ruminant populations including Djallonké sheep^19^. In consequence, we could expect that orthologous cattle chromosomal areas in which some QTLs for trypanotolerance related traits were previously identified^27^ would coincide with the genomic areas harboring genes of functional importance for adaptation in Djallonké sheep. However, only one orthologous candidate gene located on BTA4 (*CAV1*) had a clear relationship with those QTLs.

QTL information on cattle trypanotolerance was refined experimental herds^65^ and outbred populations^66,67^. Such analyses suggested that QTLs located on BTA2, BTA4 and BTA7 (with genes *ARHGAP15*^65^, *TICAM1*^65^, *CXCR4*^66^ and *INHBA*^67^ being the stronger candidates to underlie these QTLs) could have a major role on cattle trypanotolerance response. Although recent analyses failed in identifying causal mutations on the cattle *ARHGAP15, TICAM1, CXCR4* and *INHBA* genes^68–70^ the importance of BTA2, BTA4 and BTA7 for trypanotolerance cannot be neglected. The case of BTA4 would have been confirmed by the current analyses. Moreover, the sheep chromosomal areas orthologous to the QTLs reported on BTA4 (OAR4) might have been conserved between species to play a role for adaptation to the same harsh environment and trypanosome challenge.

In summary, the genome of the Djallonké sheep provided new insights on the genomic basis for adaptation. This sheep population is subject to the particularly harsh environment of the hot-humid, trypanosome challenged, West Africa. The genomic areas identified are associated with innate immunity and thermotolerance. Results further suggest that genes involved in the regulation of oxidative and metabolic stress can be target of research for the ascertainment of the genetic basis of adaptation. Moreover, our findings suggest that the functional importance of cattle BTA4 for trypanotolerant response might have been conserved between species. Furthermore, our findings highlight the importance of obtaining information from non-cosmopolite livestock populations managed in particularly harsh environments.

### Ethics statement

Blood and hair root samples used here were collected by veterinary practitioners with the permission and in presence of the owners. For this reason, permission from the Ethics Committee for Health Research in Burkina Faso (Joint Order 2004-147/MS/MESSE of May 11, 2004) was not required. In all instances, veterinarians followed standard procedures and relevant national guidelines to ensure appropriate animal care.

## Supplementary information

Supplementary Dataset 1.

## Data Availability

The dataset used and analyzed during the current study is available from the corresponding author on reasonable request.
